# Parents’ experiences following conversations about their young child’s weight in the primary health care setting: a study within the STOP project

**DOI:** 10.1186/s12889-022-13803-8

**Published:** 2022-08-12

**Authors:** Karin Eli, Catharina Neovius, Karin Nordin, Markus Brissman, Anna Ek

**Affiliations:** 1 Unit for Biocultural Variation and Obesity, School of Anthropology and Museum Ethnography, University of Oxford, Oxford, UK; 2 Warwick Medical School, University of Warwick, Coventry, UK; 3 Regional Unit for the Well-Baby Clinics, Sachsska Children’s Hospital, Stockholm, Sweden; 4 Division of Pediatrics, Department of Clinical Science, Intervention and Technology, Karolinska Institutet, Stockholm, Sweden; 5 Occupational Therapy & Physiotherapy, Allied Health Professionals Function, Karolinska, University Hospital, Stockholm, Sweden

**Keywords:** Pediatrics, Child, Parent, Weight perception, Qualitative research, Interviews, Overweight, Obesity, Primary health care, Health care communication

## Abstract

**Background:**

In primary healthcare, conversations between clinicians and parents about young children’s overweight are key to providing support and initiating weight management. However, given the sensitivity of this topic, these conversations are difficult for both clinicians and parents and are sometimes delayed or avoided. To understand the emotional impact of these conversations, this study aims to shed light on parents’ experiences following conversations with primary care nurses about their child’s overweight.

**Methods:**

Participants were recruited through a childhood obesity randomized controlled trial (RCT) conducted in Sweden. Telephone-based semi-structured interviews were conducted with 17 parents (mean age 40; the majority were women (*n* = 12/17), had a university degree (*n* = 14/17) and were born in Sweden (*n* = 13/16)). Their children were between 3 and 7 years old (mean age 4.8 years) with overweight (*n* = 7) or obesity (*n* = 10). The interviews were recorded, transcribed, and analyzed using thematic analysis.

**Results:**

Two main themes were developed. Theme 1, ‘*Receiving the overweight/obesity diagnosis*’, explores parents’ reactions to the conversation with the primary care nurse. Depending on how the nurse presented the topic, conversations either fostered an alliance between the parent and the nurse, encouraging parents to reflect and develop insights about the child’s and the family’s needs (subtheme *Conversations that empower*), or felt limited, uncomfortable, or belittling (subtheme *Conversations that provoke resistance*). Theme 2, ‘*Parenting a child with a formal diagnosis of obesity*’, explores challenges parents faced following the weight conversations, including managing their own feelings and concerns (subtheme *Fear of transferring weight anxiety*), dealing with others’ reactions (subtheme *Involve family and manage surroundings*) and asking for and receiving support from health care professionals (subtheme *Obtain support from health care professionals*).

**Conclusions:**

While conversations with primary care nurses about children’s weight were often emotional, most parents felt these conversations were ultimately helpful, as they encouraged them to enact positive lifestyle changes. Importantly, when nurses initiated conversations in a responsive, non-blaming way, inviting parents to reflect on their situation, parents felt more supported and empowered. These findings convey the importance of providing communication skills training to pediatric healthcare professionals, with particular focus on childhood overweight and obesity.

**Trial registration number:**

ClinicalTrials.gov NCT03800823; 11 Jan 2019.

**Supplementary Information:**

The online version contains supplementary material available at 10.1186/s12889-022-13803-8.

## Background

Childhood overweight and obesity pose a primary healthcare challenge. Globally, among children under the age of five years, overweight and obesity have rapidly increased, such that 39 million young children are now thought to have overweight or obesity [1]. In OECD countries, national data on the prevalence of obesity among preschoolers are still limited. However, in the US, the prevalence among 2–5-year-old children is reaching 16% [2], and in the UK, about 10% of 6-year-old children have obesity [3]. In Sweden, data from the child health care services, including height and weight measurements of 90% of 4-year-olds, show a prevalence of 9% for overweight and 2% for obesity, with a variation of 10 to 16% across different regions [4]. This prevalence is a concern as young children with obesity are likely to have obesity throughout childhood [5, 6], which is associated with immediate risk for low self-esteem and depression, as well as longer-term risks for type 2 diabetes and cardiovascular disease [7]. To prevent these risks for mental and physical health, treatment should be initiated in early childhood: the latest Cochrane reviews on treatment across all ages found that treatment is most effective at the preschool age [8]. In Sweden, conversations about children’s overweight and obesity must be initiated in the child health care (CHC) setting in order to initiate further support and referral to treatment. Sweden’s CHC settings are similar to primary pediatric care in other countries, with pediatric nurses offering regular follow-ups, support and guidance to all families of young children.

Over the years, as the prevalence of obesity among young children has increased, the responsibility of CHC nurses in the detection of childhood obesity and referral to treatment has increased in parallel [9]. However, this ​​responsibility has proven somewhat difficult to manage [10]. In Sweden, the CHC nurse follows all children’s health and growth development in regular free-of-charge health care visits from birth to the age of 5. This makes the CHC an important arena to prevent overweight in preschool-aged children [11]. Previous studies found that parents perceived CHC nurses, who often know families well and have established a trusting relationship with both children and parents, as best suited to address a child’s overweight [12–14]. Although nurses consider conversations about overweight as an important task, their confidence in speaking to parents about a child’s increasing weight is low [10, 11, 15]. In both Swedish and international studies, nurses report they lack training on childhood obesity, as well as time, staffing, and guidelines for how to initiate conversations in an appropriate way and thus enable further support and referral to treatment [10, 15–17]. Across the studies, nurses have expressed apprehension about speaking to parents, reflecting the lack of consensus about how to initiate these conversations and the points to be discussed [9]. Thus, although nurses know they should speak to parents about a child’s increasing weight status and support the family in making lifestyle changes, there is no agreed guidance on how to initiate and structure these conversations. Moreover, when nurses do initiate these conversations, they feel concerned about eroding the trusting relationships they have developed with the parents over the years [10, 11, 18]. According to the Swedish Handbook in Child Health Care, the conversations are recommended to start with the CHC nurse showing the child’s growth chart to the parents, to visualize the child’s weight development. The conversation could then continue with the nurse first asking the parents about their thoughts, and then asking if she can share her thoughts [19]. Nurses note that weight conversations can become difficult if parents do not agree with the nurse, do not find the child’s weight a problem, or do not want to discuss the child’s weight at all [15, 18]. Successful conversations, on the other hand, are those where parents had already recognized the child’s overweight, or where parents are open to what the nurse says, ask questions and want to discuss the family’s habits [10]. Best practice guidelines regarding weight conversations are under development and some practical tools have started to form for clinical use [9, 20]. This type of research is key to minimize the risk that children with overweight and obesity may not receive the health care they need [15, 16].

A further challenge is parents’ reluctance to initiate a conversation about their child’s weight, for fear of facing weight stigma socially and within healthcare settings. This encompasses parents’ concerns about how weight discussions might affect their child’s wellbeing and self-esteem, alongside concerns about being blamed for their child’s weight and being labelled a bad parent [13, 14]. However, previous qualitative research has shown that parents feel responsible in preventing and handling their child’s overweight and that regular weight checkups are important [13, 21, 22]. In an Australian study, parents of children aged 2–5 years with overweight reported feeling uncertainty and loneliness in handling their child’s weight [23]. The feeling of loneliness could be even stronger if their concern for the child’s weight was not confirmed or taken seriously by the pediatric nurse [23]. Another challenging aspect for parents of children with overweight is the balancing of obesity prevention with the promotion of a healthy body image. In Denmark, parents of children aged 3–6 years with overweight who participated in a project aiming to promote healthy habits, worried about whether there was too much focus on weight loss, and felt this focus could damage the children’s self-esteem and self-confidence [24]. However, the parents also expressed what the authors termed a ‘moral dilemma’: although concerned that treatment would lead to eating disorders or a harmful body image, they also felt treatment was important for the child’s health and self-esteem later in life [24]. An important angle on this dilemma was identified in a Norwegian study with parents of children aged 2.5–5.5 years who were classified as having overweight, where those parents who had overweight themselves favored treatment, saying they did not want their children to face the same difficulties they had been through as children [22].

The importance of *how* health care professionals provide information about children’s weight has been reported in several studies. Parents report that it is key that conversations are held in a non-blaming manner [13, 21, 22]. Parents appreciate information about future risks associated with obesity but emphasize that conversations about growth and health are better than those that focus on weight [13, 21]. This has also been recognized in a scoping review of best practices in how to talk to parents and children about overweight, where a more solution-focused conversation emphasizing health rather than weight was reported as especially helpful [9]. Solution-focused conversations with carefully chosen words encourage parental involvement and subsequent action [22, 25]. In addition to these findings, a mixed methods systematic review assessed parents’ and children’s experiences of communication in connection to routine weight screening measurements in health care and school settings [26]. The authors report on the importance of clear communication both prior and post measurements to decrease worries and anxieties (e.g., what is going to happen during the measurement, what will happen afterwards, how to read a weight chart, who will have this information) but also to guide future actions [26]. Also, the findings show that parents prefer that weighing and weight conversations take place in the health care setting rather than in the school setting [26].

Studies conducted in different national contexts have increased our understanding about how to initiate conversations with parents about their children’s high weight [9, 17, 26]. However, few of these studies have focused on the parents of preschool aged children [13, 22–24, 27]. Moreover, little is known about parents’ experiences and actions in the aftermath of these conversations. In this study, we aim to shed light on the experiences of parents of preschoolers with overweight or obesity, following conversations about their child’s weight with a CHC nurse.

## Methods

### Design

Semi-structured telephone interviews were conducted with parents of children 2–6 years of age with overweight or obesity, enrolled in a randomized controlled trial, the More and Less Europe study (ML Europe) [28]. ML Europe is conducted at Karolinska Institutet in Stockholm, Sweden, and is part of the EU-funded Science and Technology in Childhood Obesity Policy (STOP) project. The primary aim of ML Europe is to examine the effectiveness of obesity treatment in preschoolers. A further aim is to examine how to improve childhood overweight and obesity care, from recruitment to treatment and follow-up. The present study addresses this aim. ML Europe is an international study (with studies in Mallorca, Spain and Timisoara, Romania), but only families from the Swedish cohort are included in the present study. The study was approved by the Stockholm Ethical Committee (Dnr: 2018/2082–31/1, December 11, 2018) and registered at ClinicalTrials.gov January 11, 2019, NCT03800823. To ensure the quality of the reporting of our research, The Consolidated Criteria for Reporting Qualitative Research (COREQ) checklist was used.

### Participant recruitment

Eligible families (i.e., randomized families who had not yet started treatment) were recruited to the study through a letter of inquiry sent to their home by mail. The letter described the purpose of the interviews, the time required, that it was voluntary to participate and that not participating in the interviews would not affect their future participation in ML Europe. The parents were also informed that the interviews would be conducted as part of a master’s degree project by CN, a pediatric nurse with extensive experience of working as a clinician and healthcare developer within the CHC setting. Interviews were also conducted by a research assistant with a master’s degree in nutrition. The research assistant had previous experience in conducting semi-structured interviews with CHC nurses regarding conversations about young children’s weight. To follow-up on the letter of inquiry, the families were contacted by telephone to ask if they wanted to participate in the interviews. During these phone calls, information about the study was provided and any questions raised by the parents were answered. If the parents agreed to participate, the parent who had visited the CHC most was asked to participate in the interview. In families where parents shared the CHC visits equally, the parents decided who would be interviewed. The interview took place when most suitable for the parent.

### Semi-structured interviews

The individual semi-structured interviews were conducted by phone between October 2019 and January 2020. All parents were asked the same core questions. Additionally, to obtain deeper understanding, the questions were probed with follow-up questions such as: Could you describe this specific situation further? How do you mean? Can you please give me an example? At the beginning of each interview, a brief introduction was given about the purpose of the study and how the interview would be conducted; parents were also reminded then that the interview would be recorded. Verbal consent to participate was obtained before the interview started. The recorded interviews were transcribed verbatim by CN and by a registered dietitian with a degree in journalism. During the interviews short field notes were taken to remember topics for follow-up questions.

The interviews followed an interview guide that was constructed by CN in collaboration with a registered dietitian and senior researcher (AE) and, a pediatric nurse with a MSc in nursing (KN). All experts have extensive experience of talking with parents about overweight and obesity and of conducting interview studies. The questions were tested in two cognitive interviews. In cognitive interviews, the questions are systematically evaluated in order to find sources of error and to ensure that the respondents perceive the questions as intended [29]. During the cognitive interviews, the think-aloud method was used, and the interviewer used verbal probing to develop a deeper understanding of respondents’ answers. The interviewer further examined the interview guide by asking respondents how they understood the meaning of certain words used in the interview questions. For example, the interviewer asked respondents: If you were to formulate the question, how would you have formulated it? How did you arrive at this answer? Was the question easy or difficult to answer? [29]. The questions that did not capture what was intended or were difficult to understand were removed or reworded. For example, the question “Was your child in the room during the conversations? If so, how did you feel about your child being involved in the visit?” was reworded to “Was your child in the room during the conversations? If so, how was the child involved in the visit?”. Finally, some words were changed, and some supplementary questions were added to deepen and clarify the interview. The final interview guide consisted of 14 open-ended questions.

### Thematic analysis

The interviews were analyzed using thematic analysis, with a realist approach [30]. A realist approach attends to participants’ experiences, the meanings they ascribe to these experiences, and how these weave into the participants’ everyday lives [30]. The transcribed interviews were read repeatedly by CN and KN for an overall understanding of the material. CN then coded the material inductively, without being limited by predetermined codes although related to the research question [30]. Reading through the transcribed data, short notes were taken to identify overall patterns in the text. During the coding of data, relevant text was highlighted, moved into a new document, and sorted according to codes. During the whole process, CN, KN and AE had regular meetings to discuss the analysis. When the coding was complete, the codes were sorted into different themes and subthemes. After that, the process of naming the themes started. Finally, all identified themes were organized in a table with quotes, codes, and subthemes.

The study team includes five co-authors (four females and one male) from different disciplines. Four of the five co-authors have a clinical background with expertise in pediatric obesity, nursing, physiotherapy, and dietetics, of these four, three have concurrent academic appointments (a research nurse, a postdoctoral fellow, and a senior researcher). The first author is a medical anthropologist who is not a clinician.

## Results

### Sample description

Of the 28 parents who received the inquiry letter, five declined to participate and six parents did not reply. Thus, 17 parents (12 mothers and five fathers) of 17 children were interviewed. Data saturation was reached, and the sample size also met established criteria for saturation in thematic analysis studies [31]. The interviews lasted an average of 34 min (18 – 48 min). Twelve parents had a university degree and three had a foreign origin, the mean age was 40.0 years (min–max 31.5 – 48.1 years). The children’s mean age was 4.8 years (min–max 3.0 to 6.9 years), and the majority were girls (12 girls and 5 boys). All children were classified as either having overweight (seven children) or obesity (10 children), and weight status (mean body mass index standard deviation score, BMI SDS) 2.4 (SD 0.6, min–max 1.5 to 3.9) was calculated using international age and sex specific reference data [32]. Mean BMI SDS for girls was 2.4 (SD 0.7, min–max 1.6 to 3.9) and for boys 2.5 (SD 0.6, min–max 1.5 to 3.1).

**Fig. 1:**
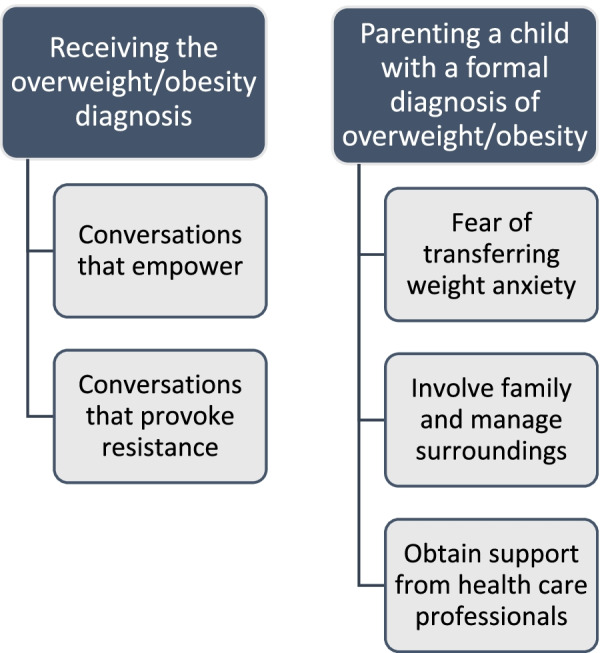
Themes and subthemes

The thematic analysis resulted in two main themes: ‘*Receiving the overweight/obesity diagnosis*’ and ‘*Parenting a child with a formal diagnosis of overweight/obesity*’. Theme 1 had two subthemes and Theme 2 had three subthemes, see Fig. 1.

### Theme 1. Receiving the overweight/obesity diagnosis

When parents were first informed by a CHC nurse about their child’s weight, they experienced this as upsetting. However, depending on how the conversation proceeded, it either fostered an alliance between the parent and the nurse, encouraging parents to reflect and develop insights about the child’s and the family’s needs (subtheme *Conversations that empower*), or felt limited, uncomfortable, or belittling, triggering frustration and even anger (subtheme *Conversations that provoke resistance*).

#### Conversations that empower

Most parents felt it was appropriate and important for CHC nurses to initiate conversations about their child’s weight, even if it was difficult to hear and difficult to handle. Several said it was an advantage that the child’s weight was acknowledged early. Parents who did not receive information about their child’s weight when their child was younger said they wished the nurse had raised the issue earlier.

Of these parents, some described their conversations with CHC nurses as particularly positive. These parents said they felt listened to and that the conversation was a dialogue, with parents given the opportunity and time to ask questions. Compared to previous conversations with the CHC nurse about other health topics, many parents felt more involved in conversations about the child’s weight. Pre-existing rapport between parents and CHC nurses helped facilitate weight-related conversations. One mother said that previous serious conversations she had had with the CHC nurses created a trusting relationship, making it easier to talk about the child’s weight. Key to the conversation, this mother said, was the nurse’s non-judgmental attitude:

*“It [the conversation] was not judgmental, it was not a lecture, it was not stressful, but I was still taken seriously, and we talked about it [the weight] properly, but there were no pointed comments. I never felt like "oh my goodness, she thinks I’m the world’s worst parent because I might have an overweight child", it was nothing like that.” (Mother, Interview a)*

Parents identified the CHC nurses’ sensitive and validating language as an important part of conveying a non-judgmental attitude. For example, a parent said they felt reassured when, after expressing their worries and asking a question, the CHC nurse prefaced her reply with* “these are common reactions and feelings, and it is common to feel that way” (Father, Interview c).* Likewise, in cases where the child was present when the nurse initiated the weight-related conversation, the parents felt it was important that the nurse normalized the situation for the child, using neutral and non-alarming language:

*“She gave our daughter something to play with and told her that “we adults are going to talk a little”, I thought she told us in a very smooth manner but also very … in the serious situation, listen[ed] and asked if we were surprised over it [the daughter’s weight]* *and if there was something regarding her weight that we had thought about.” (Mother, Interview d)*

The same mother also described how the nurse spoke sensitively, in order to prevent the child from fully understanding what had been discussed:

*“… she [the nurse] was aware not to talk about this in such a way that our daughter understood exactly what it was about, and she sort of suggested that we should take [the information home], to land [settle/take this information in], to talk a little at home and then have a meeting, a phone call together, … we could quickly agree on that.” (Mother, Interview d)*

In some cases, the child took active part in the conversation, but the focus was then on healthy habits rather than weight. Parents said that when nurses kept the conversation at the child’s level and engaged with the child, this could be a positive experience:

*“She [the child] can give feedback on what we have done and what we have talked abou﻿t*, *it has been very good actually, and I’ve never experienced that she [the child] felt … bad about it, but it has been at her level, and it has been just right and not pushed too much either.” (Mother, Interview e)*

Parents said that conversations where the nurse invited them to reflect on their child’s and family’s situation and needs were particularly constructive, with the nurse offering an empathetic ‘outsider’ voice. For example, one mother recollected:

*“I also remember that one of the first [conversations], like, when she had stated that ‘I see that a lot has happened in the last year' and asked me ‘what are your reflections, … have you reflected on this [weight change] during this year?’, she kind of invited me to think and land [settle/take this information in].” (Mother, Interview d)*

When being told about their child’s weight, some parents said the use of the child’s weight chart in the conversation helped to dedramatize but at the same time capture the seriousness of the conversation:

*“… [it became] very visual with this graph and that (the nurse said) "then you see it [the child weight development] very clearly here, but we will follow up …". So, I think it got us to notice [the child’s increased weight status] in a very good professional way.” (Mother, Interview d)*

Several parents said the conversation with the nurse led to further dialogue at home, cooperation with their partners and behavior changes. In families where parents disagreed about the child’s weight being a problem, the conversations with CHC nurses could lead to consensus-building between the parents: *“… it started a very good dialogue between me and my husband about this and how we both act in different situations and what can trigger what …, so it started a very good conversation in the family” (Mother, Interview d)*.

#### Conversations that provoke resistance

Some parents felt their CHC nurse lacked enough knowledge to facilitate in-depth discussions about the child’s weight development. These parents said their nurse initiated weight conversations but provided inadequate information, could not answer their questions, and did not provide sufficient explanations. One mother said the CHC nurse said her daughter was *“two above the curve” (Mother, Interview l)* on the weight chart but provided no further explanation. The mother had to ask, *“What does it mean? Is it dangerous? Should I do something?”* and said that *“[i]f I had not asked such questions, she might not have said more than that she is two lines above the curve” (Mother, Interview l)*. Other parents said the nurse provided information they already knew, feeling, consequently, that they were not being listened to and that the nurse did not understand the family’s needs and requests for support. As one father explained, *“The only thing that conversation was really about was that he was not allowed to eat too much sugar and he had to be more active outdoors. Yeah, no shit! That was too basic. That, we have already managed to figure out” (Father, Interview i)*. Along similar lines, a mother said, *“…there is a lot of focus on sweets … as soon as you talk about weight … [but] you feel that there are other things too” (Mother, Interview m)*.

Some parents described a lack of commitment on the part of the nurse and felt that addressing the child’s weight was a process that they themselves started and pushed. In these cases, the nurse had not seen the child’s weight as a problem in the same way the parents did, and the parents felt the nurse did not take their concerns seriously. For example, one father expressed a concern that , saying that *“… if we raise the question when we are at the CHC then she [the nurse] says. ‘Yes but try to think about eating a lot of vegetables’ … you see, there has been no more [guidance] than that.” (Father, Interview b)*.

In contrast, other parents said they experienced the weight conversation repeatedly, which led them to feel accused and attacked every time they visited the CHC. One father said:

*“If I can be blunt, it feels stupefying [the conversations at CHC]. You go in there and someone points with their finger “no, no” [the way you would say to a child], … that’s what it feels like … that you are a fool and a complete idiot that has let your child become fat …” (Father, Interview i)*

Another parent described the weight conversation as making her feel both helplessness and defensive:

*“And then there was a nurse there … who simply said, ‘he is super overweight and obese, and this is diabetes and there is no hope’, like. … So, of course that’s not nice to hear and you get offended and then you become defensive because it’s your child and such, but above all, there were no broader hints [discussions] about how big he really was. Or why he’s so big. It was just blindly looking at this number, like.” (Mother, Interview e)*

Across the interviews, parents preferred for weight-related conversations to take place when the child was not present, and some explained they wanted to protect their children from these conversations as they were still so young. When children were present during these conversations, parents felt it prevented them from* “talking freely” (Mother, Interview j)*. In some cases, parents felt the conversation made their child realize that their weight and appearance were being discussed. The father quoted above also reported that a weight-related conversation triggered body image concerns in his young son:

*“When he was drawing [during a visit to the CHC] … He was there busy drawing and then [he asked] ‘Dad, should I not do this? Did I do something wrong?’, Like something like that. And then when we got home; ‘But Dad, what is it with my stomach?’ ... So that, yes, really completely wrong!” (Father, Interview i)*

Several parents left the CHC visits feeling failure and regret, burdened by the sense they had made mistakes and feeling powerless to change the situation. One father described asking himself:* “Should you have done something else when [the child] was two? But yes, I do not know, it is easy to be wise in hindsight … You can feel this as a small failure … in some way, in terms of… what eating habits you give to the child.” (Father, Interview j)*.

Notably, feeling a lingering sense of failure was a problem unique to weight-related conversations, as the same parents said they had different experiences in visits where other topics were discussed. One parent described the difference involved:

*“… regarding the more general topics such as vaccines and how the breastfeeding works … then I have felt more involved … but this might be because there I haven’t had any problems if I compare to this weight discussion, where I feel it is a little rock that gnaws somewhere.” (Mother, Interview h)*

### Theme 2. Parenting a child with a formal diagnosis of overweight/obesity

After their children were diagnosed with overweight or obesity at the CHC, parents experienced numerous challenges. These included managing their own feelings and concerns (subtheme *Fear of transferring weight anxiety*), dealing with others’ reactions (subtheme *Involve family and manage surroundings*) and asking for and receiving support from health care professionals (subtheme *Obtain support from health care professionals*).

#### Fear of transferring weight anxiety

Most parents described being shocked when CHC nurses used words like overweight or obesity to describe their child’s weight status. These words triggered parents’ anxiety both about their child’s weight and about their own parenting and followed parents in the conversation’s aftermath. The CHC conversation was often followed by a larger focus on the child’s weight in different situations – in other healthcare settings, at home, with grandparents and at pre/school. For parents, this emergence of ‘weight talk’ was accompanied by fears of triggering or transferring weight anxiety to their young children. One parent said that *“if you talk weight then I do not think, never, that the children should hear it … it is so emotionally charged for the adults and then it becomes emotionally charged for the children and it automatically becomes something negative.” (Mother, Interview l)*.

Parents were particularly worried that conversations about overweight would lead children to develop an unhealthy relationship with food and negative body image, especially as some children were already aware that they were bigger than their friends. One mother explained:

*“This thought has really hit me, if we talk, in front of the daughter, about a high weight and being chubby, how does that stay in her head when she is 10?” (Mother, Interview h)*

In addition, parents worried that talking about food might cause the child to feel guilty in eating situations. This made it challenging for parents to talk about lifestyle changes, though they felt it was still important to encourage healthy eating:

*“You always have a fear of this … that it [conversations about weight] will cause an unhealthy … view of the whole thing … when you are four years old, you register so much. … At the same time, it is always good to have this little talk … about what is healthy … that you [the child] hear it from someone else too.” (Mother, Interview f)*

Some parents had a complicated relationship with food themselves. As children, they were subject to talk about weight and weight loss in their family of origin, which they had experienced as negative. One mother said she worried her daughter might inherit her eating disorder if exposed to weight talk:

*“I do not want her to walk the same path [as I] … I remember when people talked about me as chubby ... when I was little, it was the only thing that etched itself in my head … then you have that extra challenge that you yourself remember what it was like. Yes. And that’s why I’m so damn scared she’s going to hear anything negative that it stays with her for the rest of her life.” (Mother, Interview q)*

In parenting a child with a higher weight, several parents stressed how important it was to talk about being healthy, teach their children how to make healthy choices and avoid talking about weight*:* *“… We do not talk about her weight with her … but we work on not eating sweets for the sake of her teeth and so on. That you exercise to become strong. We do not talk about how to look and so on, not yet” (Mother, Interview c)*.

Some parents raised the issue of the stigma attached to obesity. They worried that addressing their child’s overweight in a way that the child could perceive as negative, would make the child overly critical towards their own body and lead to an eating disorder.

*“… You don’t want him to get eating disorders either. So, it’s all about, like, how do you handle that? After all, it’s a young child, how can you get something to last ... how can you help him with this without it going the other way, so to speak.” (Father, Interview j)*

Some parents said they spoke with preschool staff about the child’s weight. However, the staff did not always see the child’s weight or eating as a problem. Sometimes it was difficult for the preschool staff to limit tactfully the child’s food intake, and some parents felt their child was treated as different from other children. These parents worried about how being treated differently affected their children and expressed concern that the children would be teased, as some children had already been exposed to negative comments from friends.

*“So, she may not* *even have thought about it [being treated differently], but … it’s probably just a matter of time. There was someone in the kindergarten who had said that she had a big belly or something like that ... so I’m thinking there will be more of that.” (Mother, Interview l)*

#### Involve family and manage surroundings

After the CHC conversation, parents began sharing their child’s diagnosis of overweight or obesity with family and friends. Almost all parents said their child’s grandparents wondered aloud if their grandchild was truly overweight. Many parents felt these comments were insensitive. For some, this was an added conflict in an already strained relationship with the child’s grandparents and they preferred to restrict their involvement, while others chose to keep grandparents at arm’s length as they worried about their parenting being criticized. However, notwithstanding initial disagreements about the child’s weight, many parents said they were supported by their parents and relatives. For example, one mother said: *“… they [the family] took it very strongly, was my experience … and said like ‘yes, but of course we will support you by thinking about this too’.” (Mother, Interview d)*.

Several parents mentioned they disagreed with their child’s other parent regarding the child’s weight, and some parents felt they had been left alone with the responsibility. Some even felt discouraged by the other parent in trying to enact lifestyle changes. In some families, one parent expressed doubts about whether the child was truly overweight, suggesting it was only a CHC perception. This imbalance in the family, where one parent was more engaged than the other, was a cause of arguments and disagreements in the family.

*“… it has felt a little hard that you have to work, like, yourself and be that tough one who always has to slow down [the eating], say no and stop a little when there is “fika” [coffee break that often involves a snack such as cake, a biscuit or a sandwich] and so on.” (Mother, Interview c)*

The parents’ own childhood experiences of overweight and obesity sometimes led to different perceptions in the family of how serious the child’s weight problem was; in some cases, while one parent with a personal history of childhood overweight felt the problem was serious, the other dismissed it. Parents also described how the child’s diagnosis led them to look at their everyday lives differently, and how it affected the family’s life. One mother said: *“… now you feel like you are double checking, have we really, like, been outdoors and been properly physically active? It becomes like a family matter …” (Mother, Interview f)*.

To obtain support from others outside the family, parents had to initiate weight conversations. However, some parents felt ambivalence and guilty when speaking to friends and relatives about their children’s overweight:

*“…when I brought it [the child having obesity] up with my friends, then it really felt awful to say that about a young child … I do not know, I am very ambivalent, because I think that on the one hand; let children be … I never want anyone to say anything about my child's body in front of her and, on the other hand, I feel … it is very important to be aware and keep track of the situation and all that.” (Mother, Interview l)*

In other cases, parents tried to avoid these conversations as they felt embarrassed and worried that people may judge their parenting. This was part of a wider social silence around childhood obesity, as one father described: *“I think people dare not say anything there either. … it is sensitive, when it comes to someone’s children … the question that is raised is always … ‘Are you a bad parent or not?’.” (Father, Interview i)*.

#### Obtain support from health care professionals

Following the CHC conversation, parents felt they needed to know not only ‘*what*’ they could do for their children, but also ‘*how*’ to do it. What is considered healthy food and what is an appropriate portion size for a toddler? How and where do you set the limit? For this, parents said they needed ongoing support and advice from health care professionals: *“… we requested, like, support for us parents. How do we think about food, how do we think about exercise?… like a sounding board and information support for us parents.” (Mother, Interview d)*.

Parents also said they wanted to connect with other parents of children with overweight or obesity. One mother explained that: *“… I think you kind of get more inspired and more motivated by talking with someone who perhaps has older children who … how they have done and what they did and what they thought was good and bad and stuff like that …” (Mother, Interview k)*.

The parents described it as much more difficult than they thought it would be to handle the child’s weight. After being informed about their child’s weight status, a particular challenge parents faced was managing their child’s eating behavior, for example, coping with a child who often asks for and talks about food.

*“Because (the child) does not really have a problem with eating unhealthy foods, she only eats too much, of regular food ... So, it's not … that I give her too … or inappropriate food ... The thing is that it’s too much, she's hungry all the .... she does not feel full, I think.” (Mother, Interview q)*

Although parents were aware of these behaviors before the CHC conversation, the conversation conveyed the seriousness of the child’s weight status, prompting parents to feel they needed to manage their child’s weight better. Parents described constantly thinking about steering the child away from food situations and finding ways to reward and do activities with the child without food being involved. However, parents described everyday weight management as challenging, with very limited support from health care professionals.

Without adequate professional support, parents said their child’s rapid weight gain was especially difficult to handle.

*“At the four-year visit then, it was just ‘oh my god what are we going to do now so that he does not get childhood diabetes’. Yes, it was just terrified panic with me and his mom, like. Yes, and then it was the next talk with the dietitian, then we almost just got angry about how meaningless it was.” (Father, Interview i)*

Some parents were frustrated by not achieving the desired result after all the changes they had implemented in the child’s lifestyle. They wanted more support and access to useful tools to move forward. Several reflected on how it can be so difficult to do right, even when you know what is healthy and what is not.

*“We know what is healthy and unhealthy, we do not think McDonald’s is good for our children, but the problem is that (pause) why do we not live according to that? If we know that we have children with problems, if we know this is bad for the kids, why don't we do it right then?” (Mother, Interview l)*

Following the CHC conversation, some parents turned to additional professionals for support. For example, one parent contacted a family therapist with good knowledge of weight problems. However, several parents said they had been to see a dietician but left the visit feeling disappointed and frustrated as they had expected more help. The information they had received, often regarding what is healthy food, was already known to them. The parents instead requested support in how to handle a hungry child. One mother described:

*“I do not know what support to ask for either, we have talked [with the CHC nurse] have talked about a referral to a dietitian too, but I feel it is a bit tricky, because then it is like that you… I can feel that you are being taken for a fool, that you do not know how to eat properly ...” (Mother, Interview m)*

Despite these disappointments, parents were motivated to enact change, and several said they took every opportunity to receive support. One mother emphasized it was a parent’s responsibility to accept the help offered: *“oh my goodness I have an overweight child, it is very dangerous, and she will have lots of problems in life—I do everything I can for that not to happen and it is my responsibility”. (Mother, Interview l)*. Along similar lines, another mother said: *“We must listen to the help we can get” (Mother, Interview f)*.

## Discussion

In this interview-based study, we examined parents’ experiences following conversations with CHC nurses about their preschool-aged child’s weight. Interviewed before starting a childhood obesity RCT, all participating parents had young children with overweight or obesity. We found that parents described weight conversations with CHC nurses either as empowering or as provoking resistance. Parents described conversations as empowering when nurses opened a dialogue, inviting parents to express their needs and reflect together on how to introduce lifestyle changes. In contrast, conversations that provoked resistance were those where parents felt that the nurse provided inadequate information or portrayed their child’s condition using bleak and blaming framings, which could be characterized as stigmatizing. The effects of these conversations reverberated beyond the clinic, as parents attempted to integrate the information they had been given, leading to lifestyle changes and socio-emotional challenges. Parents often felt conflicted, wishing to enact lifestyle changes but worrying that managing their child’s weight might lead their child to develop poor body image and disordered eating. In making lifestyle changes, parents sought the cooperation of family and other people in the child’s life, although this also meant coping with minimizing or unsupportive reactions. Alongside this, parents also sought additional professional support from dieticians and therapists, but often felt the support they had been given was limited.

A key finding was that parents felt empowered by competent and empathetic health care communication, with the CHC conversation conducted as a dialogue and the nurse displaying sensitivity and engagement. Although an evidence-based best-practice for weight communication is still lacking, our findings are aligned with previous studies which showed that clinicians’ attitudes and tone during conversations about children’s weight are of paramount importance [9, 27]. Together with this earlier body of work, our findings suggest that effective conversations about children’s weight should be non-judgmental, consider parents’ needs and prior knowledge, and provide concrete and individualized advice [12, 27]. According to the participating parents, an important part of non-judgmental speech was the avoidance of loaded language about weight, a finding that echoes previous research [27]. Although, as in earlier studies, parents noted the importance of being provided clear, factual information [10, 11], and also wanted to know, as early as possible, if their child’s weight was too high [9, 13, 21], they cited words such as ‘obesity’ as problematic. A similar finding was reported by McPherson et al. 2017, who found that, while the words ‘overweight’ and ‘obesity’ were accepted in the context of describing clinical charts together with a non-judgmental approach, both parents and clinicians preferred the terms ‘weight’, ‘unhealthy weight’, ‘high BMI’ and ‘weight problem’ when speaking about individual children [9]. The authors concluded that the health care practitioner explore what terms the family use and if appropriate, use these words [9].

Many parents were concerned about how to manage their child’s weight without negatively affecting their child’s body image, eating habits and self-esteem. A similar finding was reported in a Norwegian focus group study, which found that parents worried that their children, aged 4–11 years, would feel less accepted by friends and family once lifestyle changes had been implemented, and that they may develop negative self-perception or eating disorders [33]. Likewise, a Danish interview study reported that parents expressed a dilemma, fearing that in improving their child’s physical health they were risking the child’s self-worth [24]. The worries parents expressed, both in our study and in earlier research, are likely related to obesity stigma [34]. The impact of obesity stigma on caregivers’ perceptions and practices has emerged most clearly in a US-based study, where parents and grandparents of children with overweight or obesity, aged 3–5 years, described their child or grandchild using terms such as ‘cute’, or ‘chunky’, distancing themselves from ‘obesity’ and the social stigma and bullying it implied [35]. Our findings convey the sensitive balance parents must strike when beginning to manage their child’s weight and suggest that health care professionals should be careful to steer conversations away from weight and appearance and toward health [9]. Because parents of children with overweight or obesity often experience self-stigmatization, guilt, and blame, a sensitive, health-focused communication approach may encourage parents to engage more openly with treatment [36, 37].

Parents appreciated clinical advice that was tailored to their needs and knowledge. Thus, rather than receiving generic advice focused on healthier food habits to improve energy balance (e.g., eat less sugar and more vegetables and be more physically active), parents wanted information on practical strategies to use in everyday life, for example, how to estimate an appropriate portion size and how to handle a constantly hungry child. In a previous study, we found that parents of children with obesity knew what lifestyle changes to enact but needed advice on how to implement these lifestyle changes in the family [38]. Although some CHC nurses are competent in advising parents about weight management strategies, these lie outside primary care pediatric nurses’ area of expertise [15, 18]. This underlines the importance of employing a multi-disciplinary team in the CHC, including dietitians and psychologists, to provide better care to families of children with overweight. Moreover, in addition to improving the support parents are given, our findings also suggest it is important to discuss realistic expectations for the child’s weight development and behavior change processes, to reduce frustration among parents who find lifestyle changes difficult to implement or whose efforts yield limited results.

A notable finding was that, for several parents, the CHC weight conversation was instrumental in starting a dialogue with their partners or co-parents. Following the CHC conversation, these parents reported, they began to reflect together on their child’s weight problem and search for solutions as a team. This finding is novel, as similar findings have not been reported in earlier studies focused on parents’ experiences of weight conversations in primary health care. However, in our previous study, which focused on parents’ experiences of participating in parent group-based childhood obesity treatment, parents reported that the parent group discussions helped them “to start working as a team” within the family [38]. While the CHC conversation sparked teamwork in some families, it is important to note that some parents felt unsupported, faced resistance from family members, and were left to cope with their child’s condition on their own, a finding aligned with earlier research [39, 40]. Taken together, our findings suggest that pediatric clinicians should strive to involve both parents in managing the child’s weight, as well as support parents in communicating effectively with friends and family members about their child’s weight, for example, by providing them with talking points about healthy weight and lifestyle changes.

Many of the elements of the preferred conversation style described by the participating parents can be found in motivational interviewing (MI). MI is the recommended method to use in the CHC, to help families find their own resources and strengths to enable any lifestyle changes [19]. MI is primarily about assessing whether change should occur and, if so, finding motivation to implement change, without determining how change should happen [41]. This way, MI creates a conversation that is relationship-building. While MI has been used in many studies internationally to improve conversations between parents and clinicians [9], it has yet to show effectiveness in the prevention and treatment of overweight and obesity in children. In a large Swedish prevention study on childhood obesity [42, 43], using MI in CHC did not appear to be effective in influencing the child’s weight over time [42, 43]. Similarly, a well-conducted treatment study in Italy showed that using MI had no effects long-term [44]. Still, MI includes many of the conversational elements cited as valuable by the parents who participated in the present study. This suggests that MI should be evaluated further in the childhood obesity management context, with potential adaptations to this population of patients and parents. One possible adaptation would be to introduce a weight bias reduction program for clinicians, in tandem with training in MI. Although research is still needed to establish the effectiveness of such programs, studies have suggested that weight bias reduction interventions lead to positive changes in clinicians’ beliefs [45]. This, in turn, may help clinicians in communicating with parents and addressing their concerns about obesity stigma. Another adaptation would be to train clinicians in how to explore with parents factors which might have contributed to the child’s overweight/obesity, beyond dietary intake and physical activity, including familial history, significant childhood life events, and exposure to an obesogenic environment, so that they can better support families and offer personalised advice.

### Strengths and limitations

The main strength of this study is its focus on an understudied population: parents of preschoolers with overweight or obesity. The study’s semi-structured interview design has allowed us to explore these parents’ experiences and thereby generate new findings that may inform future research questions. However, some limitations need to be acknowledged. Although we tried to recruit a heterogenous sample of parents, fewer fathers than mothers were interviewed. Additionally, most parents had a university degree and were born in Sweden, meaning that families of lower SES and migrant background were underrepresented. This can be explained, in part, by the inclusion criteria of ML Europe, which stipulated that all participants should understand and speak Swedish, to be able to participate in a comprehensive RCT with group discussion elements.

### Future studies

In ML Europe, more interview studies are planned to investigate parents’ experiences of parenting a child with obesity, as well as undergoing intensive childhood obesity treatment as a family. Additionally, we plan to explore what facilitates and what hinders families’ engagement in treatment, as well as why families decline or leave treatment. Within and beyond ML Europe, future studies should recruit families of lower socioeconomic status and families of diverse cultural backgrounds, to better understand how parents across society perceive and experience child weight and weight management. Although qualitative research on weight-related conversations is increasing, best practices for weight-related conversations in pediatric contexts are still lacking. We would encourage future studies to focus on evaluating best conversational practices, as this is urgently needed to improve weight management for children of all ages and across medical settings, including those focused on treatment for obesity, feeding disorders, and eating disorders.

## Conclusions

In this qualitative analysis of parents’ experiences following conversations with CHC nurses about their children’s overweight/obesity, we found that parents wanted to discuss their child’s weight, provided that the nurse met them in a knowledgeable, professional, and engaging way. Parents appreciated conversations in which nurses invited reflection and dialogue, while using supportive language and avoiding negatively charged words. When these criteria were not met, however, parents described feeling helpless, judged, and resistant to the message. Furthermore, while parents described weight conversations as difficult, they wished nurses would initiate these conversations as early as possible; indeed, some parents wondered whether their child’s health would have been better had the nurse initiated the weight conversation earlier. Additionally, weight conversations with CHC nurses led to changes in communication within families, prompting cooperation between parents and other relatives to facilitate the child’s weight management and implement behavioral changes as a family. The main difficulty parents associated with weight conversations was the negative impact these could have on the child’s body image, either via the conversation itself, if the child was present, or following the conversation, when parents began to implement changes for the child’s weight management. Taken together, the findings convey that weight conversations in pediatric primary care, are essential in encouraging parents of children with overweight or obesity to make positive changes. Future studies should evaluate best practices in these conversations, as a crucial component of children’s weight management and long-term mental and social wellbeing.

## Supplementary Information

**Additional file 1:** 

**Additional file 2:** 

## Data Availability

The transcripts/data of this qualitative study are not publicly available due to confidentiality agreements with the participants but are available from the corresponding author on reasonable request.

## Abbreviations

BMI SDS: Body mass index standard deviation score
CHC: Child health care
MI: Motivational interviewing
ML Europe: The More and Less Europe study
STOP: Science and Technology in Childhood Obesity Policy
