# Supplementary material for: Parents’ experiences following conversations about their young child’s weight in the primary health care setting: a study within the STOP project
Source: BMC Public Health. 2022 Aug 12;22:1540. doi: 10.1186/s12889-022-13803-8 (PMC9375316; doi:10.1186/s12889-022-13803-8)
Supplement: Supplementary file 2 — Additional file 2: [file 12889_2022_13803_MOESM2_ESM.docx]

**APPENDIX 2**

**Interview guide**

First some background questions.

Your child, is it a girl or a boy and how old is your child?

Do you have other children? How old are the children?

Who in the family has most often participated in the visits to the Child health care (CHC)?

The following questions will be about how you perceive conversations with your CHC and how you’ve been treated.

CHC strives to work with various forms of support for parents.

What support do you feel you have received from BHV?

Have there been any specific topics covered?

Can you tell us how you experienced the conversations with the CHC nurse?

Can you tell us how you felt you were treated in the conversations?

Can you tell us how you perceived the information in the conversations?

Did you receive the information you requested?

Conversation about weight

One of the CHC nurse’s tasks is to monitor the child’s weight and height development.

How have you experienced the conversations about the child’s growth development?

Did you feel involved in the conversations?

Were you more or less involved in these conversations than in other conversations at CHC?

Was your child in the room during the conversation?

If so, how did you experience it?

If so, how was the child involved in the visit?

Did you want the child to be with / not with? Why / why not?

Describe what you experienced as good in the conversations?

Do you wish the conversations had been different?

If so, how would you have liked the conversations about your child's weight to be conducted?

The decision process

Now I have a question about the More and Less study, what did you think when you received information about the study?

Can you tell us how you decided to participate in the study?

Can you tell us about discussions you may have had in the family, with friends or others, in relation to you being invited to participate in the study?

What are your expectations for your future participation in the study?

Support from other care providers

Have you had contact with other health care professionals, other than the CHC nurse regarding the child's weight, e.g. doctor or dietitian? (Before or after you joined the study?)

If yes: Did you seek this help yourself? How did you experience that support?

If not; Is there something you wished you had been offered? If so, what support would you have wanted?

Do you have something you want to add that we did not talk about in the interview?

Follow-up questions to use during the interview:

Could you develop this a bit …

Could you explain in more detail …

What do you mean when you say ...

It sounds interesting, tell me more about it!

You mentioned something about… can you tell me more about it?

How?

How do you handle this?

What have you learned from it?

Can you give an example?

Did I get it right if you mean that …

I would like to go back to what you said about…

What did that mean for you?

Can you tell me more or can you describe more?
